# Reversible High‐Affinity Binding of Coagulation Factor Xa to Zeolites Induces Accelerated Blood Coagulation

**DOI:** 10.1002/advs.202417099

**Published:** 2025-04-11

**Authors:** Chaojie Shi, Meijuan He, Hao Chen, Xuefei Wei, Liping Xiao, Xiaoqiang Shang, Yifeng Shi, Qi Wang, Lisha Yu, Jie Fan

**Affiliations:** ^1^ Key Lab of Applied Chemistry of Zhejiang Province Department of Chemistry Zhejiang University Hangzhou Zhejiang 310027 China; ^2^ College of Chemistry and Chemical Engineering Nanchang University Nanchang Jiangxi 330031 China; ^3^ Hangzhou Zeolite‐Innovation Life Science Technology Co., Ltd Hangzhou Zhejiang 310018 China; ^4^ Key Laboratory of Precision Diagnosis and Treatment for Hepatobiliary and Pancreatic Tumor of Zhejiang Province The Second Affiliated Hospital School of Medicine Zhejiang University Hangzhou Zhejiang 310009 China

**Keywords:** biorecognition, coagulation, factor Xa, protease, zeolite

## Abstract

Zeolite is recognized as an essential hemostatic material for controlling massive bleeding. Elucidating the procoagulant mechanism of zeolite is critically important, as it will facilitate the rational design of more effective zeolite‐based hemostatic materials. In this study, it is discovered an extremely strong, calcium‐dependent interaction between coagulation factor Xa (FXa) and zeolite—termed target‐specific biorecognition—that mimics the FXa/factor Va (FXa/FVa) interface formed during the natural coagulation cascade. This interaction alters the prothrombin activation pathway to a more efficient mechanism, significantly amplifying FXa activity. Notably, the complex structure and FXa activity can be reversibly modulated through Na^+^/Ca^2+^ ion exchange of zeolites, offering a novel strategy for dynamically tuning enzymatic activity. Furthermore, this protein‐zeolite based biorecognition system, mediated by reversible interactions, represents a promising biomimetic platform for regulating protein bioactivity in cell‐free applications, extending its utility beyond hemostatic material development.

## Graphical abstract

A calcium‐dependent, specific biorecognition between coagulation factor Xa (FXa) and zeolite is discovered. It alters the prothrombin activation pathway to a more efficient mechanism by mimicking the FXa/factor Va (FXa/FVa) interaction formed during the natural coagulation cascade.

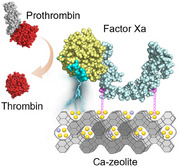

## 1 Introduction

Hemorrhage accounts for ≈40% of deaths following traumatic injury.^[^ ^1^ ^]^ The development of bioactive materials to achieve rapid hemostasis and improve survival rates in massive bleeding patients remains a critical challenge. Cellulose‐based hemostatic materials including cotton, oxidized cellulose and oxidized regenerated cellulose, have been used for decades.^[^ ^2^ ^]^ Recent innovations such as oxidized microcrystalline cellulose and SURGICEL absorbable hemostatic gauze (Johnson & Johnson) have been developed.^[^ ^3^ ^]^ Polysaccharides and their derivatives were discovered and developed as promising hemostatic agents,^[^ ^4^ ^]^ exemplified by HemCon bandage,^[^ ^5^ ^]^ and Celox granule.^[^ ^6^ ^]^ However, these materials exhibit limited efficacy in severe emergency bleeding. Although fibrin glue,^[^ ^7^, ^8^, ^9^ ^]^ a biological adhesive combining thrombin and fibrinogen, demonstrates superior hemostatic performance, its clinical application is limited by the high costs and stringent storage requirements.

Over the past two decades, natural aluminosilicate minerals, such as kaolin, zeolite and montmorillonite, have emerged as indispensable tools in civilian and military medicine for massive hemorrhage control, due to their rapid action, cost‐effectiveness, and exceptional stability.^[^ ^10^, ^11^, ^12^ ^]^ Kaolin‐based hemostatic dressings are recommended for the U.S. Department of Defense by the Committee on Tactical Combat Casualty Care (CoTCCC). In 2002, the granular zeolite hemostat QuikClot (Z‐Medica) was approved by the U.S. Food and Drug Administration (FDA) and used in the wars in Iraq and Afghanistan.^[^ ^13^ ^]^ However, these two materials always face the potential risk of their particulate matters entering the human body. In 2019, a tightly bonded and flexible zeolite‐cotton hybrid hemostat was developed, which exhibited superior procoagulant activity over kaolin‐based hemostats in a lethal femoral artery injury rabbit model and eliminated the risk of entering the body.^[^ ^14^ ^]^ Recently, a kaolin‐zeolite composite gauze demonstrated synergistic efficacy by combing their procoagulant mechanisms.^[^ ^15^ ^]^ Despite clinical adoption, the mechanistic understanding of these inorganic hemostats remains incomplete, hindering rational design.

The prevailing hypothesis attributes zeolite's procoagulant activity to blood factor concentration via water absorption and calcium ion release.^[^ ^16^ ^]^ While this explains basic interactions, it oversimplifies the process and fails to account for the superior activity of protein corona‐zeolite composite.^[^ ^17^ ^]^ Our recent work revealed that calcium‐ion‐exchanged zeolite (Ca‐zeolite) accumulates clotting factor Xa (FXa) and factor Va (FVa) to form a surface‐bound prothrombinase complex, functioning as an “inorganic platelet” to generate thrombin with unprecedented stability and activity compared to natural platelet‐based coagulation.^[^ ^18^ ^]^ This discovery advanced understanding of the procoagulant mechanism of zeolite. However, the molecular basis of the biorecognition, assembly and activation of the clotting factors on the zeolite surface has remained unclear.

Herein, we identify a high‐affinity, target‐specific interaction between FXa and Ca‐zeolite Y (CaY), which significantly accelerates procoagulant reactions. This calcium‐dependent interaction—confirmed by molecular dynamics (MD) simulations and multi‐amino acids labeling techniques—arises from covalent bonding between zeolite‐bound calcium ions and epidermal growth factor‐like domain (EGF‐LD) of the FXa light chain. The FXa/FVa‐like interface between FXa serine protease (SP) domain and zeolite surface significantly amplifies FXa activity. Remarkably, Na^+^/Ca^2+^ ion exchange reversibly modulates this target‐specific interaction, enabling tunable enzymatic activity. FXa, functioning as critical role at the intersection of intrinsic and extrinsic coagulation pathways,^[^ ^19^ ^]^ serves as a molecular target for CaY's biorecognition mechanism. This specific interaction enables CaY to recruit, assemble, and accelerate FXa activity, thereby establishing it as a determinant of hemostatic efficacy. Beyond hemostatic materials design, this reversible covalent protein‐zeolite interaction represents a biomimetic platform for regulating protein bioactivity.

## 2 Results and Discussion

### 2.1 Biorecognition of Factor Xa by Ca‐Zeolite Y

**Figure 1:**
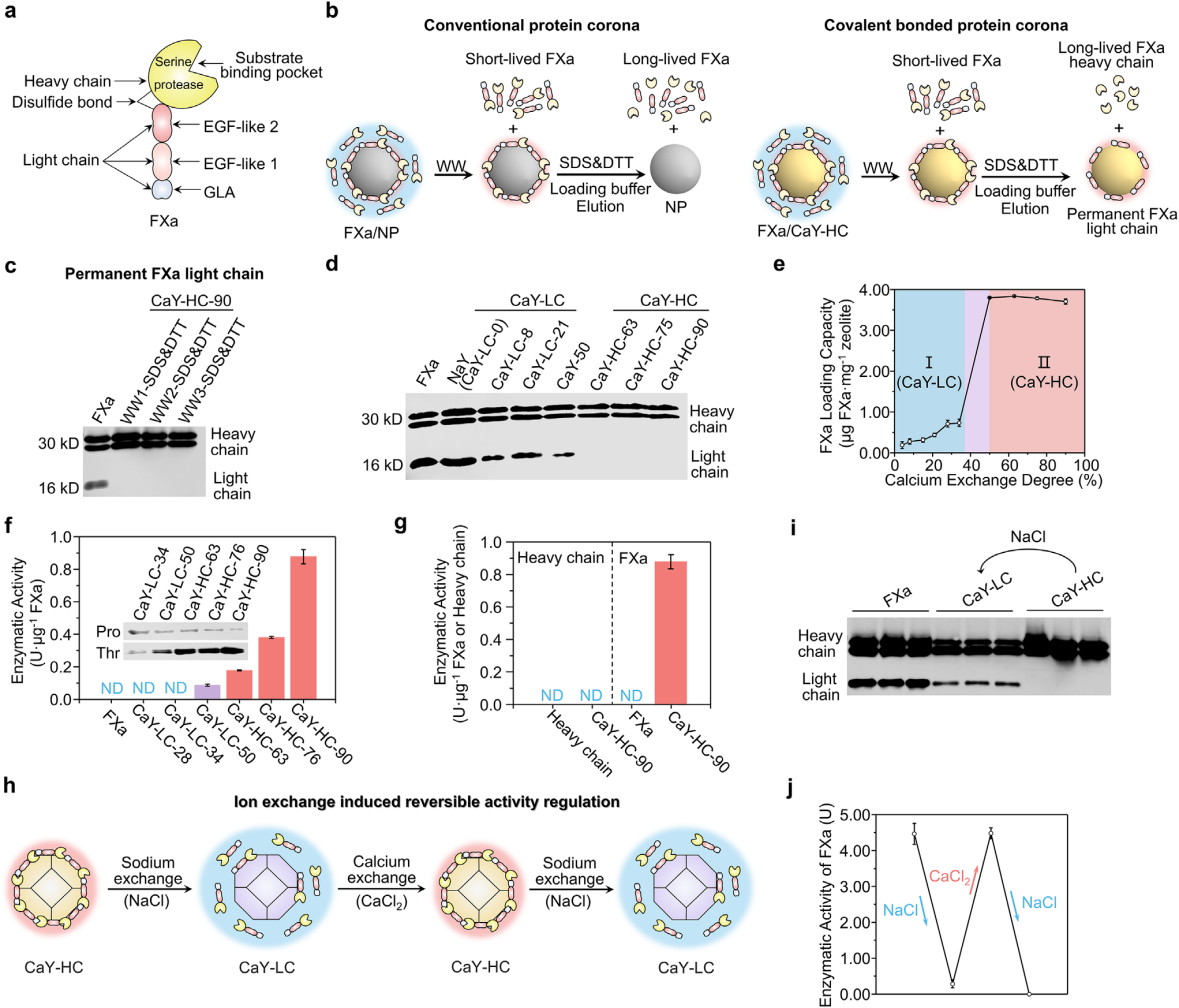
Covalently binding of FXa with CaY zeolites and the corresponding significant amplification and regulation of FXa enzymatic activity. a) Structure of coagulation FXa. b) Schematic representation of the conventional protein corona and covalent bonded protein corona, and demonstrating the bonding ability for short‐lived, long‐lived and permanent FXa. c) Bonding capacities of permanent FXa light chain with CaY‐HC‐90 zeolite via WB. d) Bonding capacities of CaY zeolites with FXa light chain via WB. e) Bonding capacities of CaY zeolites with FXa via BCA protein assay. Region I: FXa weakly bonded to CaY‐LC zeolite surface. Region II: FXa strong covalently bonded to CaY‐HC zeolite surface (n = 3). f) Specific enzymatic activity of FXa on CaY zeolites with several calcium exchange degree for prothrombin‐to‐thrombin conversion (n = 3). Pro: prothrombin; Thr: thrombin; ND: not detected. g) Enzymatic activity of FXa heavy chain, FXa, heavy chain/CaY‐HC‐90 and FXa/CaY‐HC‐90, respectively (n = 3). h) Schematic diagram of regulating FXa activity on zeolite surface via sodium/calcium ion exchange. i) Regulation of the interaction between FXa light chain and zeolite surface via sodium/calcium ion exchange. j) Enzymatic activity of FXa on zeolites via sodium/calcium ion exchange for prothrombin‐to‐thrombin conversion (n = 3). Data values correspond to mean ± SD.

Ca‐zeolite Y (CaY) samples with different calcium exchange degrees were prepared from commercial Na‐zeolite Y (NaY) via ion exchange (Tables S1–S5 and Figures S1 and S2, Supporting Information). Incubation of coagulation factor Xa (FXa, **Figure** **1**a) with these CaY materials resulted in FXa/zeolite complexes, as evidenced by significant FXa adsorption from solution. This phenomenon is consistent with the reported protein‐nanoparticle (NP) interactions.^[^ ^20^, ^21^, ^22^ ^]^ When the CaY zeolite with low calcium exchange degree, denoted as CaY‐LC, was used as NP, short‐lived FXa and long‐lived FXa were both detected from the adsorbed FXa in the FXa/zeolite complex (Figure 1b), which is classified according to the strength of the interactions as in literatures.^[^ ^20^, ^21^, ^22^ ^]^ The short‐lived FXa refers to the bonding capacity of FXa that can be directly isolated from the FXa/zeolite complex by simple water wash (WW) due to that the only weak interaction formed between them. While, the long‐lived FXa means that it forms strong but not covalent interaction with CaY‐LC zeolite, consequently, it can't be isolated by water wash but can be denatured and dissociated from the complex by a sodium dodecyl‐sulfate polyacrylamide gel electrophoresis (SDS‐PAGE).^[^ ^23^, ^24^ ^]^

Notably, CaY with high calcium exchange (CaY‐HC) exhibited a subset of FXa resistant to SDS‐PAGE (Figure 1b; Figures S3 and S4, Supporting Information), indicating covalent binding. In the post‐treatment analysis, it was found that only the heavy chain of the FXa was detected in the loading buffer solution after the SDS‐PAGE treatment. Since the SDS‐PAGE can cut the disulfide bond between the light chain and the heavy chain of the FXa,^[^ ^25^, ^26^, ^27^ ^]^ the above results reveal the formation of covalent protein corona in FXa/CaY‐HC complex is attributed to that only the light chain of the permanent FXa is covalently binds to the CaY‐HC zeolite (Figure 1c). On the contrary, the heavy chain only forms non‐covalent bonds with the CaY zeolite, because it can be removed from the complex after the disulfide bonds between the heavy chain and light chain is cut by the SDS‐PAGE treatment. Our results demonstrate that a rarely reported permanent protein corona formation is observed in the FXa/CaY‐HC complex, and the covalent bonding is only formed between the light chain of the FXa and high‐calcium zeolites.^[^ ^28^, ^29^, ^30^, ^31^ ^]^

When the CaY zeolite was replaced by other inorganic NP materials with similar compositions and structures, it was found that kaolin (a layered aluminosilicate mineral), and CuY zeolite (zeolite Y with copper ion instead of calcium ion), only form the short‐lived FXa in the FXa/NP complex (Figure S5, Supporting Information). After WW treatment, all the short‐lived FXa was separated from these NPs, resulting in no detectable protein in loading buffer solution. For SiO_2_ and amorphous silica‐alumina with calcium ion exchange (CaASA), following SDS&DTT and loading buffer isolation, long‐lived FXa was separated from the materials and it was detected in the loading buffer solution (Figure S6, Supporting Information). However, no permanent FXa was found on all of these materials, despite they have similar chemical composition or similar crystal structure as CaY zeolite. These results indicated that both the crystal topology and zeolite‐bonded calcium ions are critical in mediating the specific and covalent interactions.

The formation of permanent FXa in FXa/CaY complexes displays a calcium‐dependent manner. After the FXa was incubated with a set of CaY zeolites of different calcium exchanging degree (0%–90%) and washed following the same procedure, the FXa in the loading buffer solution was carefully analyzed (Figure 1d). Both of light and heavy chains of FXa were observed for FXa/CaY‐LC zeolites (CaY with low calcium exchange degree of 0%–40%), while only heavy chain of FXa was identified for FXa/CaY‐HC zeolites (CaY with high calcium exchange degree of 50%–90%), indicating permanent FXa was formed on these CaY samples with high calcium exchange degree. This reveals a sharp transition from long‐lived to permanent nature of FXa with increasing calcium exchange degree, and 50% calcium exchange degree seems to be a distinguishing threshold. In addition, the sharp transition of FXa binding‐types is accompanied by a striking increase in its loading capacity (three‐fold, Figure 1e), which can be attributed to the covalently‐binding pattern formed between light chain and CaY‐HC zeolites greatly increase the FXa absorption.

### 2.2 Biorecognition Amplifies and Regulates the Enzymatic Activity of Factor Xa

The enzymatic activity for prothrombin‐to‐thrombin conversion of FXa immobilized on CaY‐LC and CaY‐HC zeolites was evaluated and compared to free FXa (Figure 1f). The obtained results indicate that the covalent interaction between FXa and CaY zeolites has profound impacts on its bioactivity. Free FXa and FXa/CaY‐LC complexes possess no detectable enzymatic activity, whereas FXa/CaY‐HC complexes exhibit distinguishable catalytic activity, indicating calcium ion plays an indispensable role. The enzymatic activity significantly increases from 0.18 ± 0.003 to 0.88 ± 0.044 U µg^−1^ FXa, when the calcium exchange degree of the zeolite increases from 63% to 90%. This result demonstrates that the enzymatic activity of FXa can be adjusted by calcium exchange degree. Control experiments confirmed minimal activity enhancement for short‐lived (kaolin, CuY) and long‐lived (SiO₂, CaASA, NaY) FXa complexes, highlighting the necessity of covalent FXa‐CaY‐HC binding for activity amplification (Figure S7, Supporting Information). It is noteworthy that free FXa, FXa heavy chain, FXa heavy chain/CaY‐HC‐90 complex also exhibit no detectable enzymatic activity under physiological concentration (10 µg mL^−1^), whereas the FXa/CaY‐HC‐90 presents a significant enhanced enzymatic activity. It can be explained by that the heavy chain of FXa, in absence of the light chain, cannot covalently bind to the CaY‐HC zeolite, resulting in no obvious regulation of enzymatic activity (Figure 1g).

Furthermore, the kinetic parameters of prothrombin‐to‐thrombin conversion by FXa and FXa/CaY‐HC‐90 have been determined (Figure S8, Supporting Information). When FXa was absorbed on CaY‐HC‐90 zeolite, the maximum reaction rate V_max_ of thrombin formation represented 9‐fold increase (35.5 vs. 3.75 mol min^−1^·mol·FXa^−1^), compared to the free FXa. This significant enhancement seems to mimic the prothrombinase complex assembly of FXa and cofactor FVa onto the membrane phospholipid, which also significantly amplifying the enzymatic activity of FXa.^[^ ^31^ ^]^

The calcium‐determined covalent binding interaction not only amplifies the enzymatic activity of FXa on CaY‐HC‐90 surface, but also allows to reversibly modulate the enzymatic activity of FXa (Figure 1h). The Na^+^/Ca^2+^ ion exchange cycling experiment demonstrated that the enzymatic activity of FXa can be reversibly modulated (Figure 1i). A calcium to sodium ion exchange process disrupts the covalently binding between the FXa and Y type zeolites, reducing the enzymatic activity for prothrombin‐to‐thrombin conversion. Whereas, a sodium‐to‐calcium ion exchange process reconstructs the strong interaction between FXa and Y type zeolite, restoring excellent enzymatic activity for prothrombin‐to‐thrombin conversion. This Na^+^/Ca^2+^ ion exchange process can be continuously repeated and the enzymatic activity is adjusted accordingly without any irreversible loss, indicating it's a highly reversible behavior (Figure 1j). This is a rare phenomenon with significant implication for dynamic enzyme regulation.

### 2.3 Bio‐Inorganic Covalent Interaction Induces the Biorecognition

**Figure 2:**
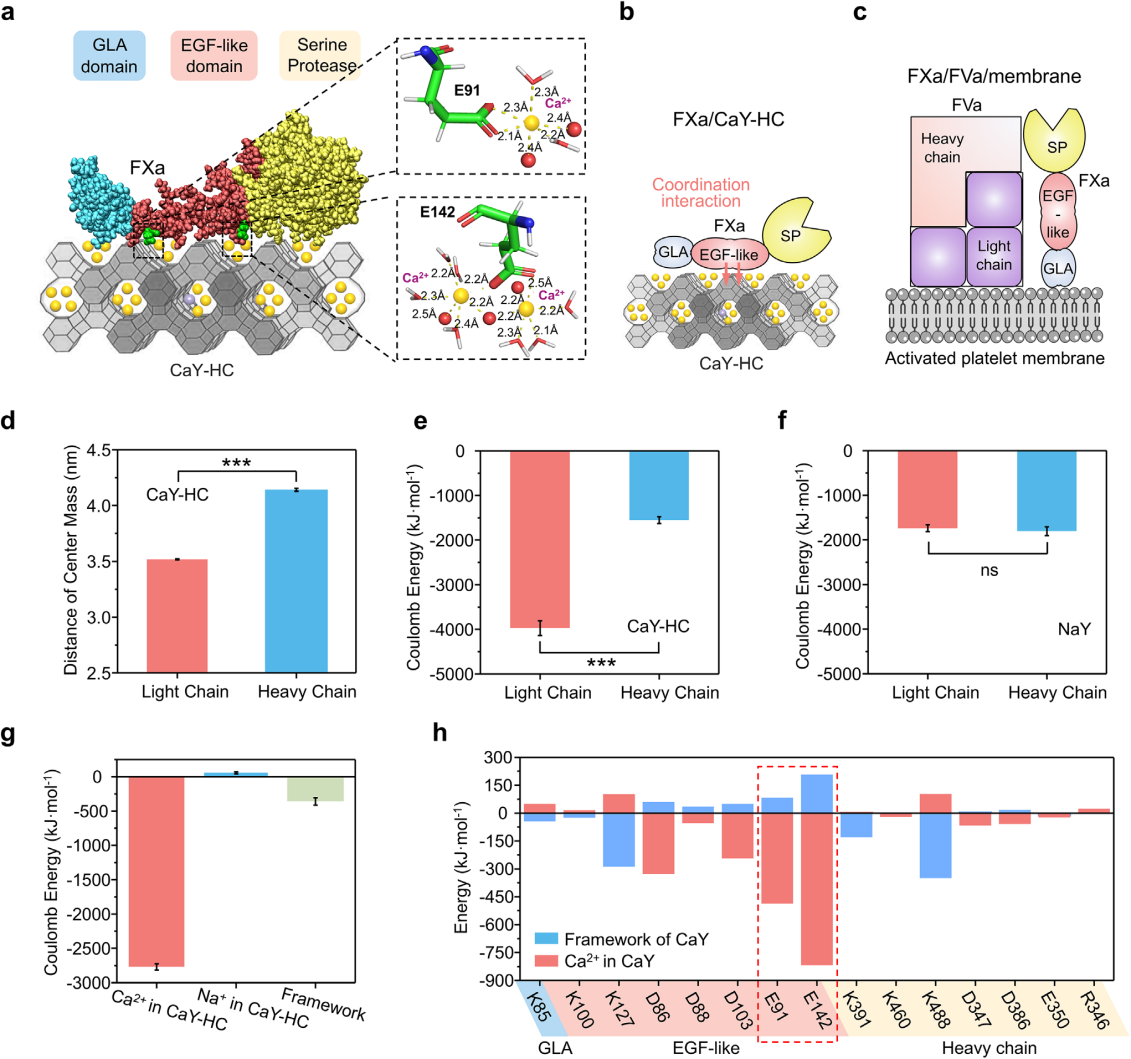
EGF‐LD‐target covalent‐binding of FXa on the CaY‐HC zeolite identified by molecular dynamics (MD) simulation. a) Conformation of FXa confined on CaY‐HC zeolite and details of E142 and E91 interacting with CaY‐HC zeolite. FXa heavy chain is shown in pale yellow, EGF‐LD of FXa light chain is shown in salmon and GLA domain of FXa light chain is shown in slate blue. b) Schematic diagram of FXa on CaY‐HC zeolite. FXa bonding on the CaY‐HC zeolite via coordination interaction of light chain EGF‐LD. c) FXa/FVa complex on the activated platelet membrane surface. d) Distance of center mass between FXa chains and CaY‐HC zeolite (n = 3). e) Coulomb energy between FXa chains and CaY‐HC zeolite (n = 3). f) Coulomb energy between FXa chains and NaY zeolite (n = 3). g) Coulomb energy between FXa light chain and the components of CaY‐HC zeolite (n = 3). (h) Binding energy between the main amino acid residues of FXa and CaY‐HC zeolite. Data values correspond to mean ± SD. Each group contains three points at energy equilibration. ns: non‐significant, ***P < 0.001, one‐way analysis of variance (ANOVA).

To further elucidate the calcium‐dependent covalent binding mechanism between the FXa light chain and the CaY‐HC zeolites, molecular dynamics (MD) simulation was employed to investigate the binding mechanism (**Figure** **2**). The overall conformation of FXa adsorbed on the CaY‐HC zeolite via MD simulation is shown in Figure 2a. The light chain contains a γ‑carboxyglutamate‐rich (GLA) domain and two epidermal growth factor‐like domain (EGF‐LD), while the heavy chain includes a serine protease (SP) domain with a catalytic triad (Figure 1a). The strong coordination interaction between FXa EGF‐LD and CaY‐HC zeolite induces a “lie down” configuration on the zeolite surface (Figure 2b). In natural prothrombinase FXa/FVa complex on activated platelet, the GLA domain of FXa light chain insets into platelet membrane, and “stands” on the activated platelet membrane parallel to FVa (Figure 2c).^[^ ^32^, ^33^ ^]^ Even though FXa displays different conformations between biological interface (FXa‐platelet) and non‐biological interface (FXa‐zeolite), these two systems share two critical features: 1) strong light chain‐interface interaction, and 2) catalytic heavy chain distancing from the interface to maximize enzymatic activity.

In detail, the distance of center mass between FXa light chain and CaY‐HC zeolite was much shorter than that of the FXa heavy chain (Figure 2d; Figure S9a, Supporting Information). The coulomb energy analysis validated that the coulomb energy between FXa light chain and CaY‐HC zeolite was significantly stronger than that between FXa heavy chain and the zeolite (3973 ± 166 vs. 1553 ± 73 kJ mol^−1^, Figure 2e; Figure S9b–c, Supporting Information). These results are consistent with the results derived from the SDS‐PAGE treatment experiment: only the light chain of FXa forms covalent bonding with CaY zeolite. In contrast, the coulomb energy between FXa light chain and NaY zeolite was comparable to that between FXa heavy chain and the zeolite (Figure 2f; Figure S10a–d, Supporting Information).

MD simulation further identifies that both of the negatively charged zeolite framework and positively charged cation can interact with FXa, while binding energy mediated by calcium ions in CaY‐HC zeolite contributes 87% of the total coulomb energy (Figure 2g). Glutamate 91 (E91) and glutamate 142 (E142) consisting of carboxyl groups, belonging to EGF‐LD in FXa light chain, contribute the strongest binding energy between light chain and CaY‐HC zeolite (Figure 2h). In consideration to binding energy (> 450 kJ mol^−1^) and binding length (< 2.5 Å), the interaction between EGF‐LD of FXa light chain and CaY‐HC zeolite is belonged to coordination interaction (Figure 2a,h).^[^ ^34^ ^]^ These results provide molecular‐level validation of covalent‐binding between EGF‐LD of FXa and CaY‐HC zeolite.

**Figure 3:**
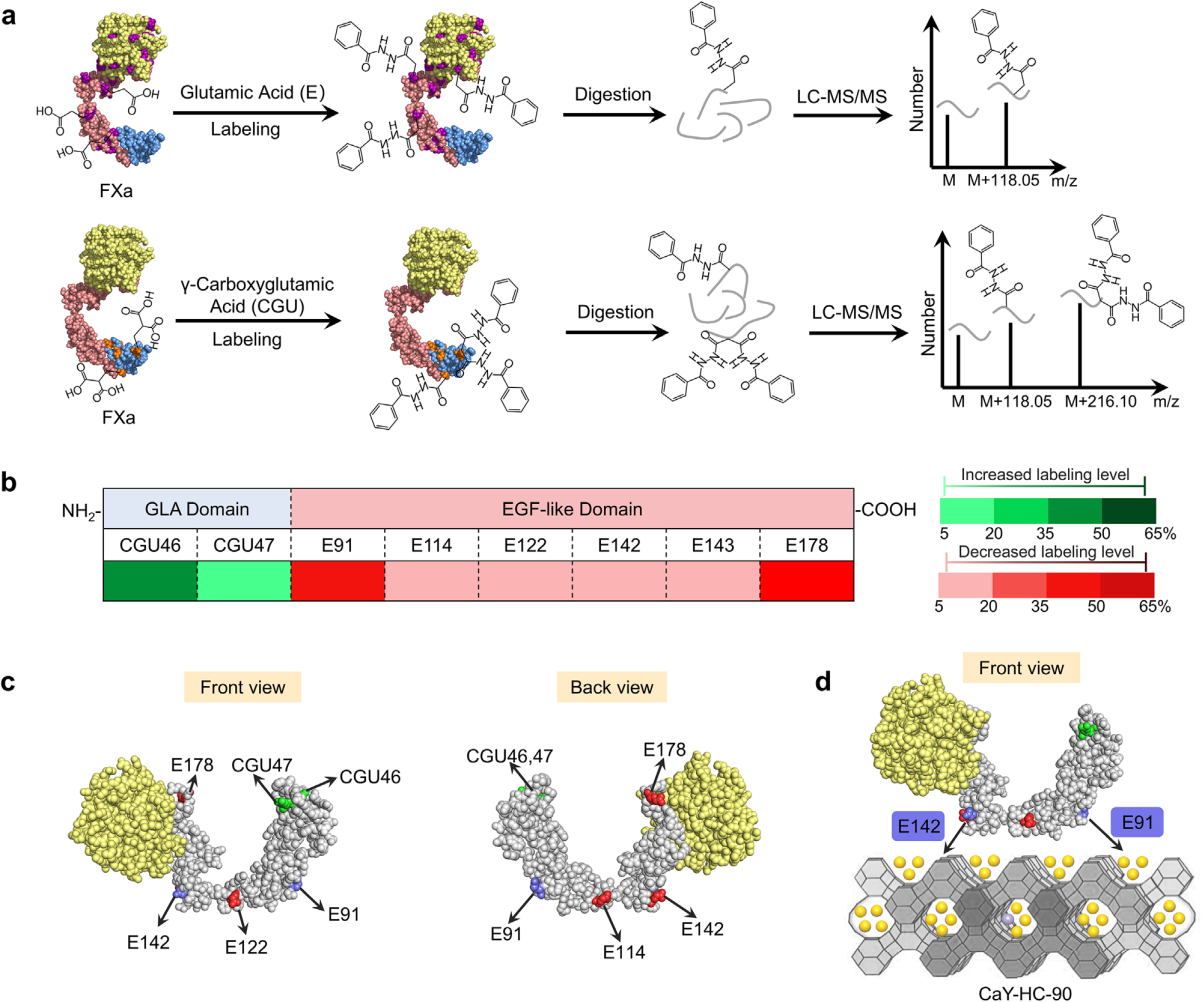
EGF‐LD‐target covalent‐binding of FXa on the CaY‐HC zeolite identified by liquid chromatography‐tandem mass spectrometry (LC‐MS/MS) analysis. a) Schematic diagram of benzoyl hydrazide (BHD) labeling for glutamate (E) and gamma‐carboxyglutamic acid (CGU) followed by mass spectrometry analysis. b) Color gradient diagram of labeling level for E and CGU residues in FXa light chain; increased labeling level is shown in green, decreased labeling level is shown in red. c) Distribution of the key amino acid residues in FXa light chain which labeling level decreased/increased in front/back view; the amino acid residues with increased labeling level are shown in green, the amino acid residues with decreased labeling level are shown in red. E91 and E142 from EGF‐LD, which strongly interact with CaY‐HC‐90 zeolite, are marked in purple. d) Target covalent‐binding conformation of FXa on CaY‐HC‐90 zeolite.

The EGF‐LD‐target covalent‐binding of FXa to CaY‐HC zeolite was further confirmed via benzoyl hydrazide (BHD) labeling glutamate (E) and gamma‐carboxyglutamic acid (CGU) followed by mass spectrometry analysis (**Figure** **3**a and Table S6, Supporting Information). Amino acid residues E and CGU consist of carboxyl groups. Free amino acid residues are more easily labeled than those engaged in the strong interaction with zeolites.^[^ ^35^, ^36^, ^37^, ^38^ ^]^ A significant reduction of BHD labeling in FXa light chain EGF‐LD is observed (Figure 3b), and the correspondent amino residues with decreased labeling level are shown in red (Figure 3c). The predicted binding sites E91 and E142 via MD simulation are also identified to decreased labeling levels. And E114, E122 and E143 exhibit decreased labeling levels, which are surrounded by E91 and E142. The CGU 46 and CGU47 in FXa light chain GLA domain demonstrate increased labeling levels (Figure 3b), suggesting no steric hindrance. A predicted conformation of FXa is shown on the CaY‐HC‐90 (Figure 3c,d), confirming that the specific interaction of FXa light chain EGF‐LD and CaY‐HC zeolite leads to biorecognition of FXa, consistent with both experimental and simulation data.

### 2.4 Biorecognition Constructs the Factor Xa/Factor Va‐Like Interface and Amplifies the Activity of Factor Xa

**Figure 4:**
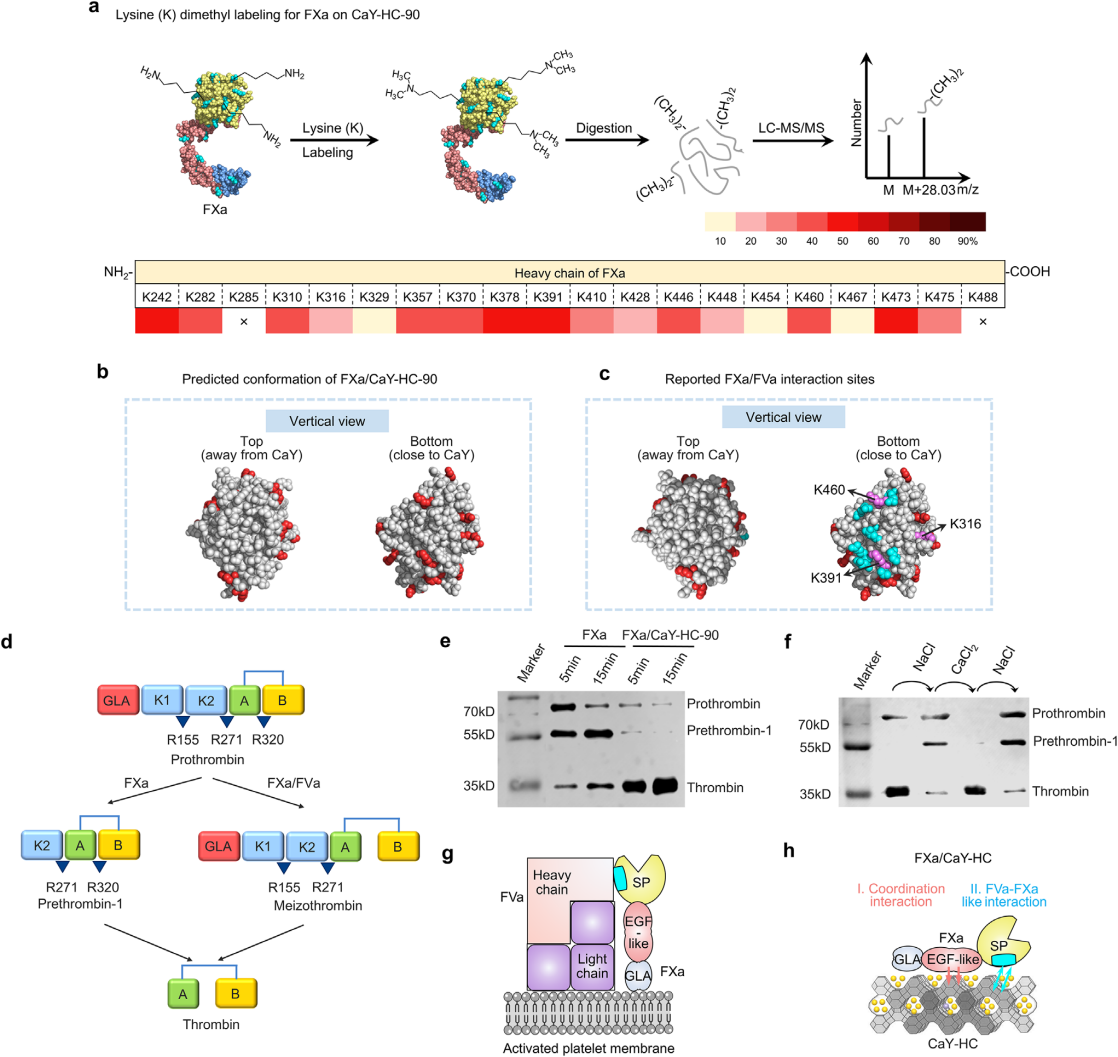
FXa/FVa‐like interface of FXa SP domain (heavy chain) on CaY‐HC zeolite and enzymatic activity of FXa/CaY‐HC for prothrombin‐to‐thrombin conversion pathway. a) Schematic diagram of lysine (K) dimethyl labeling followed by mass spectrometry analysis. Color gradient diagram of changes in amino acid residues labeling level of FXa heavy chain. Decreased labeling level is shown in red. b) Distribution of the key amino residues in FXa heavy chain of which labeling level is decreased in top/bottom view; the amino residues with decreased labeling level are shown in red. c) Distribution of the key amino residues in reported FXa/FVa interaction sites; the non‐lysine residues are colored in cyan. The lysine residues with reduced labeling in FXa/CaY‐HC‐zeolite are highlighted in purple. d) Schematic diagram of prothrombin‐to‐thrombin conversion pathway of FXa/FVa complex and free FXa. e) Prothrombin‐to‐thrombin conversion pathway activated by free FXa and FXa/CaY‐HC‐90 identified using WB. f) Prothrombin‐to‐thrombin conversion pathway activated by FXa/FVa‐like interface of FXa SP domain (heavy chain) on zeolites via sodium/calcium ion exchange. g) FXa/FVa complex on the activated platelet membrane surface with the labeled interaction domain (cyan color). h) Schematic diagram of FXa on CaY‐HC zeolite. FXa bonding on the CaY‐HC zeolite via coordination interaction of light chain EGF‐LD and FVa/FXa‐like interface of SP domain.

To understand how the EGF‐LD‐target covalent‐binding pattern of FXa induces amplified bioactivity, we investigate the conformation of FXa heavy chain, which contains the SP domain as catalytic active site, and its interface with CaY‐HC zeolite using the lysine dimethyl labeling technique combined with LC‐MS/MS (**Figure** **4**a and Table S7, Supporting Information). We observe that the dimethyl labeling levels of lysine (K) residues on one side of the heavy chain for the FXa on CaY surface are significantly reduced, compared to the heavy chain of free FXa. On the contrary, there is no obvious decrease in the labeling levels on the opposite side of the FXa heavy chain. This labeling‐reduced side of FXa heavy chain is considered to be the contacting side engaged in the bio‐inorganic interfaces (Figure 4b). The main amino acid residue contacts (< 5 Å) of FXa/FVa complex between FXa heavy chain and cofactor FVa in natural prothrombinase complex are colored in cyan.^[^ ^33^ ^]^ The K sites among these main molecular contacts exhibit significant dimethyl labeling reduction, which are colored in purple (Figure 4c). These main molecular contacts in prothrombinase complex are distributed in the contacting side between FXa heavy chain and CaY‐HC‐90 zeolite, suggesting an FXa/FVa‐like interface is constructed in FXa/CaY‐HC‐90 complex.

In addition, we find that the FXa/FVa‐like interface in FXa/CaY‐HC complex leads to a different pathway for prothrombin‐to‐thrombin conversion compared to free FXa. The prothrombin‐to‐thrombin reaction was monitored via SDS‐PAGE followed by WB. In the prothrombin activation by free FXa, prethrombin‐1 is main product formed during the initial stages of steady–state, whereas thrombin is the main end product when activated by prothrombinase (FXa/FVa complex, Figure 4d).^[^ ^39^, ^40^ ^]^ This direct activation pathway significantly increases thrombin generation rate. The main end product in FXa/CaY‐HC is thrombin (Figure 4e), illustrating that the FXa/CaY‐HC mimics the prothrombin activation pathway from prothrombinase and thereby activates prothrombin more efficient compared to free FXa. The sodium/calcium ion exchange processes can disrupt or reconstruct this FXa/FVa‐like interface of SP domain (heavy chain) on zeolites (Figure 4f), thereby regulating the enzymatic activity for prothrombin‐to‐thrombin conversion. The same activation pathway indirectly proves the existence of the FXa/FVa‐like interface in FXa/CaY‐HC, which plays a critical role in significant amplification of FXa enzymatic activity (Figure 4g,h).

As one of the largest classes of enzymes, proteases catalyze the hydrolysis of peptide bonds within proteins and play critical roles in diverse biological processes, including development, differentiation, cell migration, blood coagulation, and immunity.^[^ ^41^, ^42^ ^]^ The regulation of their biocatalytic functions involves reversible associations of proteases with cell membranes, which are responsible for initiating and terminating biochemical processes. In blood coagulation process, several key coagulation proteases (clotting factors) participate in the blood coagulation cascade. Following traumatic injury, activated platelet membranes rapidly externalize phosphatidylserine to the outer leaflet, providing binding sites for these coagulation proteases in the cell‐based model.^[^ ^43^ ^]^ As a critical “gatekeeper” at the intersection of intrinsic and extrinsic coagulation pathways, Factor X (FX) becomes activated to FXa through proteolytic cleavage.^[^ ^44^, ^45^ ^]^ FXa subsequently acts as an active serine protease and a component of the macromolecular prothrombinase complex, alongside FVa, phospholipid, and calcium ions. Optimal FXa enzymatic activity requires biorecognition between its γ‐carboxyglutamate‐rich (GLA) domain and the activated platelet membrane. This biorecognition not only enhances FXa activity^[^ ^46^, ^47^, ^48^ ^]^ but is also essential for successful prothrombinase complex assembly,^[^ ^46^ ^]^ which regulates hemostasis and prevents life‐threatening blood loss.^[^ ^10^, ^49^ ^]^

In this work, we demonstrate that CaY zeolite, as an inorganic material, mimics both activated platelet membranes and FVa by bio‐recognizing FXa, modulating its conformation, and amplifying its enzymatic activity. Crucially, this enhancement arises from a bio‐inorganic interface between FXa and zeolite that mimics the FXa/FVa interaction in the prothrombinase complex. MD simulations, catalytic activity measurements, and prothrombin‐to‐thrombin conversion pathway analyses, collectively demonstrate that the conformation and bioactivity of FXa can be similarly regulated by inorganic CaY zeolite.

This mimicry of physiological system by zeolite is consistent with the inorganic platelet activity of zeolite, which we previously report.^[^ ^17^ ^]^ Furthermore, this biomimetic biorecognition is attributed to covalent binding between FXa EGF‐LD and zeolite‐bound calcium ions, enabling continuous and reversible regulation through ion exchange. This spontaneous bio‐inorganic covalent interaction may provide new perspective for enzyme immobilization, suggesting that inorganic materials could biomimetically modulate protein bioactivity as observed in natural processes. Meanwhile, given that the excellent prothrombin‐to‐thrombin conversion capability and favorable biosafety (Figures S11 and S12, Supporting Information), the FXa/CaY‐HC‐90 complex can be an effective hemostatic material potentially and makes it promising to address life‐threatening hemorrhage.

## 3 Conclusion

In summary, we demonstrated that FXa protein can be anchored on CaY‐HC zeolite surface via a high‐affinity and specific coordination interaction between the EGF‐LD of FXa light chain and the zeolite‐bound calcium ions. The topology and calcium ions of CaY‐HC zeolite play critical roles in mediating this EGF‐LD‐target covalent‐binding interaction. This interaction is essential for constructing an FXa/FVa‐like interface between the SP domain (heavy chain) and CaY‐HC zeolite, which not only shifts the prothrombin activation pathway toward a more efficient approach, but also significantly amplifies the FXa enzymatic activity. Moreover, ion exchange enables continuous and reversible regulation of the EGF‐LD‐target interaction and the FXa/FVa‐like interface on the zeolite, providing a novel strategy to modulate enzymatic activity. This discovery advances the understanding of zeolite‐mediated coagulation mechanism and design of novel hemostatic materials. The unique protein‐zeolite biorecognition via reversible covalent bonding may serve as a promising biomimetic strategy to regulate the bioactivity of other proteins, particularly in cell‐free applications beyond the development of hemostatic materials.

## Conflict of Interest

The authors declare no conflict of interest.

## Supporting information



Supporting Information

## Data Availability

The data that support the findings of this study are available on request from the corresponding author. The data are not publicly available due to privacy or ethical restrictions.
