# Supplementary material for: Reversible High‐Affinity Binding of Coagulation Factor Xa to Zeolites Induces Accelerated Blood Coagulation
Source: Adv Sci (Weinh). 2025 Apr 11;12(22):2417099. doi: 10.1002/advs.202417099 (PMC12165020; doi:10.1002/advs.202417099)
Supplement: Supplementary file 1 — Supporting Information [file ADVS-12-2417099-s001.pdf]

ADVANCED SCIENCE
Open Access

Supporting Information

for *Adv. Sci.*, DOI 10.1002/advs.202417099

Reversible High-Affinity Binding of Coagulation Factor Xa to Zeolites Induces Accelerated Blood Coagulation

Chaojie Shi, Meijuan He, Hao Chen, Xuefei Wei, Liping Xiao, Xiaoqiang Shang, Yifeng Shi, Qi Wang*, Lisha Yu* and Jie Fan**

Supporting Information

Reversible High-Affinity Binding of Coagulation Factor Xa to Zeolites Induces Accelerated Blood Coagulation

Chaojie Shi, Meijuan He, Hao Chen, Xuefei Wei, Liping Xiao, Xiaoqiang Shang, Yifeng Shi, Qi Wang*, Lisha Yu*, Jie Fan**

C. J. Shi, M. J. He, H. Chen, X. F. Wei, L. P. Xiao, Y. F. Shi, Q. Wang, L. S. Yu, J. Fan

Key Lab of Applied Chemistry of Zhejiang Province,

Department of Chemistry, Zhejiang University,

Zhejiang, Hangzhou, 310027, China

E-mail: yifengshi@zju.edu.cn, qiwang@zju.edu.cn, lishayu@zju.edu.cn, jfan@zju.edu.cn.

L. S. Yu

Key Laboratory of Precision Diagnosis and Treatment for Hepatobiliary and Pancreatic Tumor of Zhejiang Province,

The Second Affiliated Hospital, School of Medicine, Zhejiang University,

Zhejiang, Hangzhou, 310009, China

Y. F. Shi

Hangzhou Zeolite-Innovation Life Science Technology Co., Ltd,

Zhejiang, Hangzhou 310018, China

X. Q. Shang

College of Chemistry and Chemical Engineering, Nanchang University,

Jiangxi, Nanchang, 330031, China

Experimental Section

Materials

Commercial zeolite Y (Si/Al = 1.98) were supplied by Sigma-Aldrich (334448). Human Factor Xa (ab62229, Abcam). Human Factor Xa Heavy chain (ab80019, Abcam). Loading buffer (LC2570, Thermo). 4 × nonreducing sample buffer (LC2570, Thermo). Dithiothreitol (D121076, Aladdin). Calcium chloride (10005861, SCR). Sodium chloride (10019318, SCR). Micro BCA Protein Assay Kit (23235, Thermo). Human Factor Xa primary antibody (ab89334, Abcam. MA1-43014, Thermo). Fluorescent secondary antibody, Anti-mouse IgG (H+L) (DyLightTM 800, Cell Signaling Technology), Anti-rabbit IgG (H+L) (DyLightTM 800, Cell Signaling Technology). Sodium cyanoborohydride (S105661, Aladdin). Formaldehyde (10010061, SCR). EDC (PG82079, Thermo). Benzoic hydrazide (B13071, Sigma). Human Prothrombin (ab62536, Abcam). Thrombin Chromogenic substrate (CS-38(01), HYPHEN BioMed). Human Thrombin (20408ES80, Yeasen Biotech Co., Ltd.). Acetic acid (10000218, SCR). Trypsin, Proteomics Grade (T6567, Sigma). Coomassie Blue Super Fast Staining Solution (P0017F, Beyotime).

Methods

Preparation of CaY zeolites

Add 3 g NaY zeolite to 15 mL calcium chloride solution with different calcium ion concentrations and stir at room temperature for 24 h. Then centrifuge to remove the supernatant, and wash the obtained solid product with ultrapure water, then dried at 80 °C. The obtained CaY zeolites with different calcium concentrations were named according to their calcium exchange degree. The calcium concentration of CaY zeolites was identified by inductively coupled plasma optical emission spectrometer (ICP-OES). Add 3 g of NaY zeolite to 15 mL 5 M calcium chloride solution and stir at room temperature for 24 h, then centrifuge to remove the supernatant, add 15 mL of 5 M calcium chloride solution again, and stir at room temperature for 24 h, repeat for three times. Then the zeolite was washed by ultrapure water and dried at 80 °C. The calcium exchange degree of CaY zeolite was calculated as:

$$\text{Calcium exchange degree} = \frac{n_{Ca} * Z_{Ca}}{n_{Ca} * Z_{Ca} + n_{Na} * Z_{Na}}$$

Characterizations of CaY zeolites

Calcium concentrations analysis. The Ca^{2+} exchange degree was measured by inductively coupled plasma optical emission spectrometer (720ES(OES), Agilent).
X-ray diffraction. The X-ray diffraction (XRD) patterns were recorded on a Rigaku Ultimate IV with Cu Kα radiation (20 °/min). The accelerating voltage and the applied current were 40 kV and 30 mA, respectively.
Transmission Electron Microscopy. TEM images were recorded on a transmission electron microscope (HT7700, Hitachi) with an acceleration voltage of 100 kV.
Laser Diffraction Particle Sizing and Zeta Potential. Size distribution and zeta potential were measured by a laser diffraction nanoparticle size potential analyzer (ZEN3600, Malvern).
NH_3-Temperature Programmed Desorption. The temperature-programmed desorption of ammonia (NH_3-TPD) was conducted on a Microtrac BELCat II instrument. 100 mg of sample was pretreated in helium at 300 °C for 1 h to remove adsorbed impurities. After the temperature cooled to 50 °C, the catalyst was exposed to an ammonia flow (5% ammonia in He, 30 mL/min) for 1 h. Physiosorbed ammonia was removed for 30 min under a flow of helium (30 mL/ min), and then the temperature was increased from 50 to 800 °C at a ramp rate of 10 °C/min.
Pyridine infrared spectroscopy analysis. Pyridine infrared spectroscopy (Py-IR) was measured on infrared spectrometer (PerkinElmer Frontier), with a spectral resolution of 4 cm^{-1}. The samples were dehydrated in an IR cell for 2 h under vacuum prior to pyridine adsorption. Py-IR spectra was measured at ambient temperature after desorption at 200 °C.

Preparation of Calcium exchanged amorphous silica-alumina (CaASA).

Amorphous silica-alumina was synthesized by a hydrothermal method. Briefly, the molar composition of the gel used was $NaAlO_2$: SiO_2: NaOH: TEAQH; H_2O=1: 25: 0.4: 9: 416. The solution of sodium aluminate, sodium hydroxide, and tetraethylammonium hydroxide were mixed at room temperature with magnetic stirring for 1 h. Then, colloidal silica was added and stirred for 1 h at room temperature. After aged for 12 h, the solution was hydrothermally treated at 150 °C for 10 h. The solid product was collected and washed with ultrapure water by suction filtration until the pH value of filtrate neutral. Then the collected product was transformed by ion exchange using a 0.5 mol/NH_4Cl solution at 85 °C and repeated three times. The obtained solid product was washed three times with ultrapure water and ethanol by suction filtration, dried at 100 °C and calcined in a muffle furnace at 550 °C for 6 h. The obtained ASA was calcium exchanged using 5 M calcium chloride solution at room temperature for 24 h. Repeat three times. Then centrifuge to remove the supernatant, and washed the obtained solid product with ultrapure water, then dried at 80 °C.

Preparation of copper ions exchange of zeolite Y (CuY).

Add 3 g NaY zeolite to 15 mL saturated copper chloride solution and stir at room temperature for 24 h. Repeat three times. Then centrifuge to remove the supernatant, and wash the obtained solid product with ultrapure water, then dried at 80 °C.

Mass spectrometry analysis.

The mass spectrometry analysis was performed based on our previous work. Briefly, 0.1% (v/v) formic acid (FA)/H_2O and FA/acetonitrile (ACN) were used as mobile phase A and mobile phase B, respectively. Separation gradient: solvent B 3 to 8% in 10 min, 8-20% in 110 min, 20-90% in 23 min. MS parameters of LTQ Orbitrap mass spectrometer (Thermo Fisher, Orbitrap Elite): ion transfer capillary 250 °C, spray voltage 1.8 kV, and full MS scan from m/z 400 to 2000 with a resolution of 60000 FWHM in centroid mode. The sequences of FXa were obtained from UniProt (P00742). Peptides were processed using trypsin cleavage, and up to 3 missed cleavage sites were allowed. Peptide mass tolerance was 10 ppm, and fragment mass tolerance was 0.05 Da. The amino acid sequence of the FXa was identified by using PEAKS Studio 8.0.

Preparation of FXa/zeolite complexes.

Add zeolites to the FXa solution and incubated at room temperature for 30 min. Subsequently, centrifuge the mixture and wash the resulting solid product three times with ultrapure water.

Bonding capacities of CaY zeolites with FXa via Western Blot.

2% SDS, concentrated 4 × nonreducing sample buffer (LC2570, Thermo) and DTT (final concentration 100mM) was added to FXa/zeolite composites to recover FXa from the zeolites. The sample were heated at 70 ºC for 5 min to denature and strip off FXa from zeolite. As a reducing agent, DTT can cleave the disulfide bond and isolate the light chain and heavy chain of FXa before running them on SDS-PAGE gel. Then, the samples were centrifuged at 20000 rpm for 10 min and the obtained supernatants were loaded and ran on SDS-PAGE gel. Primary antibody: Anti-Factor X antibody (ab89337, Abcam. MA1-43014, Thermo). Secondary antibody: Anti-mouse IgG antibody (Anti-mouse IgG(H+L) DyLight™ 800 4X PEG Conjugate, CST).

Western Blot.

The SDS-PAGE gel was running at 100 V for 2 h. The gel was transferred to a polyvinylidene difluoride (PVDF) membrane at 100 V for 1 h. The primary antibody was incubated overnight at 4 °C. The secondary antibody was incubated at room temperature under light protection condition. The membrane was imaged using an Odyssey

CLx Imager (LI-COR).

Analysis of FXa loading rate on CaY zeolite with different calcium exchange degree.

CaY zeolites (5 mg) with different calcium exchange were added 200 μL of the corresponding FXa solution for incubation at 37 °C for 30 min. Then centrifuged and took the supernatant, and identify the amount of FXa using Micro BCA Protein Assay Kit (23235, Thermo).

Specific enzymatic activity of FXa on CaY zeolites identified by thrombin chromogenic substrate assay.

10 mg CaY with different calcium exchange degree were incubated with 150 μL 40 μg/mL FXa solution at 37 °C, respectively. Then centrifuged samples at 20000 rpm for 10 min to remove the supernatants. Samples was washed with ultrapure water for three times, and the obtained pellets were re-suspended in 50 μL ultrapure water. 50 μL 0.5 mg/mL human prothrombin solution were added and incubated at 37 °C for 10 min. Then 50 μL 5 mg/mL thrombin chromogenic substrate (CS-01(38), HYPHEN) was added and incubated at 37 °C for 5 min. After that, 50 μL acetic acid was added to terminated the reaction. Samples were centrifugated at 20000 rpm for 10 min to obtain the supernatants. The absorbance of the supernatants was determined at 405 nm (Multiskan FC, Thermo). The corresponding absorbance of human thrombin (20408ES80, Yeasen Biotech Co., Ltd.) was used to form a standard curve.

Conversion of prothrombin to thrombin.

50 μL of human prothrombin (0.5 mg/mL) was added to FXa/zeolite complexes. After reacting for different periods, the reaction was stopped by adding 60 μL of 4X nonreducing fluorescence compatible loading buffer (LC2570, Thermo) and boiled at 70 °C for 7 min. The protein mixture was then analyzed in 10% reducing SDS-PAGE and western blot. Primary antibody: Anti-thrombin antibody (ab17199, Abcam). Secondary antibody: Anti-mouse IgG antibody (Anti-mouse IgG(H+L) DyLight™ 800 4X PEG Conjugate, CST).

Calculation of kinetic parameters.

The Michaelis-Menten kinetic constants Km and Vmax for free FXa and FXa adsorbed on CaY-HC-90 zeolite surface were calculated from the Lineweaver-Burk plot. Thrombin production after 15 min was determined for reactions using 50, 100, 200, 300, 400, 500, 600 μg/mL of human prothrombin for free FXa. Thrombin production after 13.25 min was determined for reactions using 50, 100, 200, 300, 400, 500, 600 μg/mL of human prothrombin for FXa adsorbed on CaY-HC-90 zeolite.

Quantitative thrombin generation.

Thrombin recognizes the cleavage site of the thrombin chromogenic substrate (H-D-Phe-Pip-Arg-pNa, 2HCl) (CS-01(38), HYPHEN). After cleaving, the absorbance of the released chromophore pNa at 405 nm is positively corelated with enzymatic activity thrombin. 50 μL of human prothrombin (0.5 mg/mL) was added to FXa/zeolite complexes, and the system reacted for 10 min. Then 50 μL of chromogenic substrate (5 mg/mL) were added. The reaction was conducted at 37 °C for 5 min, then 50 μL of acetic acid was quickly added to stop the reaction. The absorbance of the supernatant was determined at 405 nm (Multiskan FC, Thermo). The corresponding absorbance of human thrombin (20408ES80, Yeasen Biotech Co., Ltd.) was used to form a standard curve.

Molecular dynamics simulation.

The molecular dynamics simulation was performed base on our previous work. Briefly, the starting structure of FXa was taken from the complete cryo-electron microscope structure reported[1]. The zeolite unit cell with composition Al56Si136O384 (Si:Al = 2.5:1) was used, constructed based on the FAU zeolite included in the Materials Studio 8.0 package and the zeolite model consists of 4×4×1 cells in the x, y and z directions, respectively. Na^+ and water molecules were added to neutralize the charge of the system, and part of Na^+ was replaced by Ca^{2+} to obtain CaY zeolite with 40% ion exchange degree. Following the metal cation equilibration, most of metal cations on the zeolite surface was Ca^{2+}, which was used as CaY-HC zeolite to performance the interaction of CaY-HC zeolite and FXa. Before models (CaY-HC zeolite and FXa) were combined into one system, separate equilibrations were first performed. The models were solvated in TIP_3P water and counter-ion tanks respectively and underwent 20,000 steps of energy minimization. Then these systems were equilibrated for 5 ns under NVT MD runs. After the models were equilibrated, the FXa were put close to the CaY-HC zeolite surface and ensure that there is enough space between the protein and surface. In this case, the heavy or light chain are simultaneously close to the surface of zeolite. After equilibration, 200 ns production runs with NVT ensemble were carried out. All simulations in this work were carried out by GROMACS 5.0.7 package with a time step 2 fs.

Lysine dimethyl labeling and glutamic acid benzoyl hydrazide labeling reactivity profiling.

For the protein adsorbed on the nano-zeolite, the conformational changes and binding sites of the protein can be analyzed by measuring the protein lysine microenvironment. The entire experimental procedure is mainly divided into dimethyl labeling, benzoyl hydrazide labeling, eluting from CaY-HC-90 zeolite, enzymatic hydrolysis, and mass spectrometry analysis.

(i) Benzoyl hydrazide (BHD) labeling. The CaY-HC-90 zeolite (100 mg) was incubated with FXa (100 μg) in 1 mL ultrapure water at 37 °C for 60 min. Then centrifuged to remove the supernatant, washed by ultrapure water 3 times and resuspend in 200 uL water. Next, analyzed the protein concentration on CaY zeolite using BCA Protein assay (P0011, Beyotime) and diluted the solution to 0.55 mg/mL. Then, BHD (26.35 mg/mL) and EDC (7.4 mg/mL) were added, the final concentrations of two reagents in the solution were 20 mM and 500 μM respectively. The reaction was conducted at 37 °C for 20 min. To stop the reaction, 20 μL ammonium acetate (1 M) were added, final concentration was 50 mM, and incubated for 20 min. Dimethyl labeling. The CaY-HC-90 zeolite (100 mg) was incubated with FXa (100 μg) in 1 mL ultrapure water at 37 °C for 60 min. Then centrifuged to remove the supernatant, washed by ultrapure water 3 times and resuspend in 200 μL water. Next, analyzed the protein concentration using BCA Protein assay (P0011, Beyotime) and diluted the solution to 0.1 mg/mL. Then, $NaBH_3CN$ (0.6 M) and CH_2O (4%) were added, the final concentrations of two reagents in the solution were 5 mM and 10 mM respectively. The reaction was conducted at 37 °C for 25 min. To stop the reaction, NH_4HCO_3 (5 M) were added, final concentration was 500 mM, and incubated for 20 min.

(ii) Eluting from CaY-HC-90 zeolite. After labeling, the sample was washed by ultrapure water for 3 times. Then saturated sodium chloride solution was added and incubated at room temperature for 24 h. Next, centrifuged and removed the supernatant, 2% SDS and 5 × reduced loading buffer were added, and boiled at 100 ºC for 7 min. Then performed SDS-PAGE.

(iii) Enzymatic hydrolysis. Cut the target bands (approximately 1 mm^3) with a scalpel blade and place into centrifuge tubes. Added 200-400 μL 100 mM NH_4HCO_3/30% ACN for decolorization, washed until transparent. Then removed the supernatant and freeze drying to remove decolorizing liquid. Added 90 μL of 100 mM NH_4HCO_3 and 10 μL of 100 mM DTT to each tube, and incubated at 56 °C for 30 min to reduce the protein. Removed the supernatant, added 100 μL 100% ACN to each tube, and aspirated after 5 min. Added 70 μL of 100 mM NH_4HCO_3 and 30 μL of 200 mM IAA to each tube (prepared freshly, store in the dark) for 20 min. Removed the supernatant, added 100 μL of 100 mM NH_4HCO_3 to each tube, and incubated at room temperature for 15 min. Removed the supernatant, added 100 μL 100% ACN to each tube, aspirated after 5 min, and lyophilized. After freeze-drying, added 50 μL of 2.5 ng/μL trypsin solution to each tube and placed it in a 4 °C refrigerator for 60 min to allow the gel block to fully swell. Then added 50 μL of 50 mM NH_4HCO_3, incubated at 37 °C for 20 h. Next, aspirated the liquid and transferred into a new tube. Added 100 μL 60% ACN/0.1% TFA to the original tube and sonicated for 15 min. Aspirated the solution and merged it into the previous solution. Repeated the extraction 3 times, combined and lyophilized.

Regulation of enzymatic activity of FXa on CaY zeolites.

CaY-HC-50 zeolite (80 mg) was dispersed in 800 μL 300 μg/ mL FXa solution. After stirring, the mixture was incubated at 37 °C for 60 min, and the FXa was absorbed to the surface of the CaY-HC-50 zeolite. After incubation, centrifuged and removed the supernatant, and washed by ultrapure water for 3 times. Subsequently, 800 μL ultrapure water was added and well mixed. Took out 100 μL to perform Western Blot and quantitative thrombin generation assay. The remaining suspension is centrifuged to remove the supernatant. Then 700 μL 0.4 M sodium chloride solution was added and incubated at room temperature for 10 hours. Subsequently, replaced the supernatant with 700 μL of water, took out 100 μL to perform Western Blot and quantitative thrombin generation assay. The remaining suspension is centrifuged to remove the supernatant and 600 μL 0.4 M calcium chloride solution was added and incubated at room temperature for 10 h. Repeat this five times. Ensure the ratio of solution used for ion exchange to CaY-HC-50 zeolite was maintained at 100 μL/10 mg for each incubation process.

Regulation of covalently binding interaction between FXa on CaY zeolites.

CaY-HC zeolite was incubated with FXa solution at 37 °C to obtain the FXa/CaY-HC complex. A part of the FXa/CaY-HC complex was re-suspended in saturated sodium chloride solution to perform the sodium exchange. The sodium exchange treatment was performed at room temperature for 24 h. 2% SDS, 1 M DTT and non-reducing sample buffer were added to the samples before and after sodium exchange. All samples were heated at 70 °C for 5 min. Then centrifuged and the obtained supernatants were analyzed by SDS-PAGE and western blot. Primary antibody: Anti-Factor X antibody (ab89337, Abcam. MA1-43014, Thermo). Secondary antibody: Anti-mouse IgG antibody (Anti-mouse IgG(H+L) DyLight™ 800 4X PEG Conjugate, CST).

Cytotoxicity study.

The cytotoxicity studies of CaY-HC-90 and FXa/CaY-HC-90 are conducted by using Cell-Counting-Kit-8 (CCK-8, C0038, Beyotime) and Calcein/PI Cell Viability/Cytotoxicity Assay Kit (C2015M, Beyotime), respectively. In detail, FXa/CaY-HC-90 complex was prepared by adding 100 mg CaY-HC-90 into 1000 μL 50 μg/mL FXa (ab62229, Abcam) solution and incubated at 37 ºC for 40 min. After incubation, centrifuge the sample at 20000 rpm for 10 min, the obtained pellet was FXa/CaY-HC-90 complex. FXa/CaY-HC-90 complex was resuspended in 10 mL minimum essential medium (MEM) and incubated at 37 ºC for 24 h. The CaY-HC-90 extract was obtained by using the same method. For CCK-8 assay, cells (L929) were dispersed in 100 μL MEM or extracts and cultured at 37 ºC for 24 h. Then 10 μL CCK-8 solution was added and cultured at 37 ºC for 30 min. The absorbance was measured at 450 nm using a microplate reader (Vario skan LUX, Thermo). For Calcein/PI cell viability/cytotoxicity assay, L929 cells were dispersed in 200 μL MEM or extracts and cultured at 37 ºC for 24 h. Then the MEM or extracts were removed and washed with 200 μL phosphate buffered saline. 200 μL Calcein/PI solution was added and cultured at 37 ºC for 30 min. Live cells were imaged using a laser confocal microscope (Leica) at excitation/emission wavelengths of 490 nm/515 nm, while dead cells were imaged at 535 nm/617 nm.

Statistical analysis.

The experimental data were articulated as mean values ± standard deviation (SD). Sample size (n) for each statistical analysis was indicated in figure legend. Comparison between groups was made using analysis of variance (ANOVA). P-value of less than 0.05 was considered statistical significance. All statistical analyses were performed using Origin software.

Table S1. Elemental composition of NaY zeolite

Sample	Si/ Al	Na (wt. %)	Ca (wt. %)
NaY	1.98	6.66	0.03

Table S2. Calcium content of CaY zeolites detected using by inductively coupled plasma-optical emission spectrometer (ICP-OES)

Sample	Ca^{2+}-exchanging process[a]	Calcium concentration (wt %)	Ca^{2+} exchange degree
CaY-LC-4	0.01 M $CaCl_2$ (24 h RT)	0.24	4%
CaY-LC-8	0.02 M $CaCl_2$ (24 h RT)	0.46	8%
CaY-LC-15	0.04 M $CaCl_2$ (24 h RT)	0.89	15%
CaY-LC-21	0.06 M $CaCl_2$ (24 h RT)	1.25	21%
CaY-LC-28	0.08 M $CaCl_2$ (24 h RT)	1.61	28%
CaY-LC-34	0.1 M $CaCl_2$ (24 h RT)	1.97	34%
CaY-50	0.2 M $CaCl_2$ (24 h RT)	2.93	50%
CaY-HC-63	0.5 M $CaCl_2$ (24 h RT)	3.69	63%
CaY-HC-75	1 M $CaCl_2$ (24 h RT)	4.36	75%
CaY-HC-76	5 M $CaCl_2$ (24 h RT)	4.40	76%
CaY-HC-90	5 M $CaCl_2$ (24 h RT repeated three times)	5.26	90%

[a] Ca^{2+} exchanging concentration and processing time of each procedure.

Table S3. Textural property of NaY and CaY-HC-90 zeolites

Samples	Surface Area[a] (m^2/ g)	Pore Volume[b] (cm^3/ g)	Pore Width[c] (nm)
NaY	672.863	0.260	0.718
CaY-HC-90	609.569	0.244	0.718

[a] Surface area was determined by the BET equation.

[b] Pore volume was calculated by the DFT method.

[c] Pore width was calculated by the DFT method.

Table S4. NH_3 temperature programmed desorption (NH_3-TPD) analysis of NaY and CaY-HC-90 zeolites

Sample	Start temp. (℃)	End temp. (℃)	Temp. width (℃)	Area (count)	mmol/ g
NaY	50	800	750	488.02	7.257
CaY-HC-90	50	150	100	72.72	1.081
	150	800	650	447.49	6.654

Table S5. Determination of acidic sites on the surface of NaY and CaY-HC-90 zeolites determined via pyridine-infrared spectroscopy (Py-IR)

Sample	Bronsted acid (μmol/g)	Lewis acid (μmol/g)	Total acid (μmol/g)	B/L
NaY	7.51	77.02	84.53	0.098
CaY-HC-90	440.67	46.30	486.97	9.52

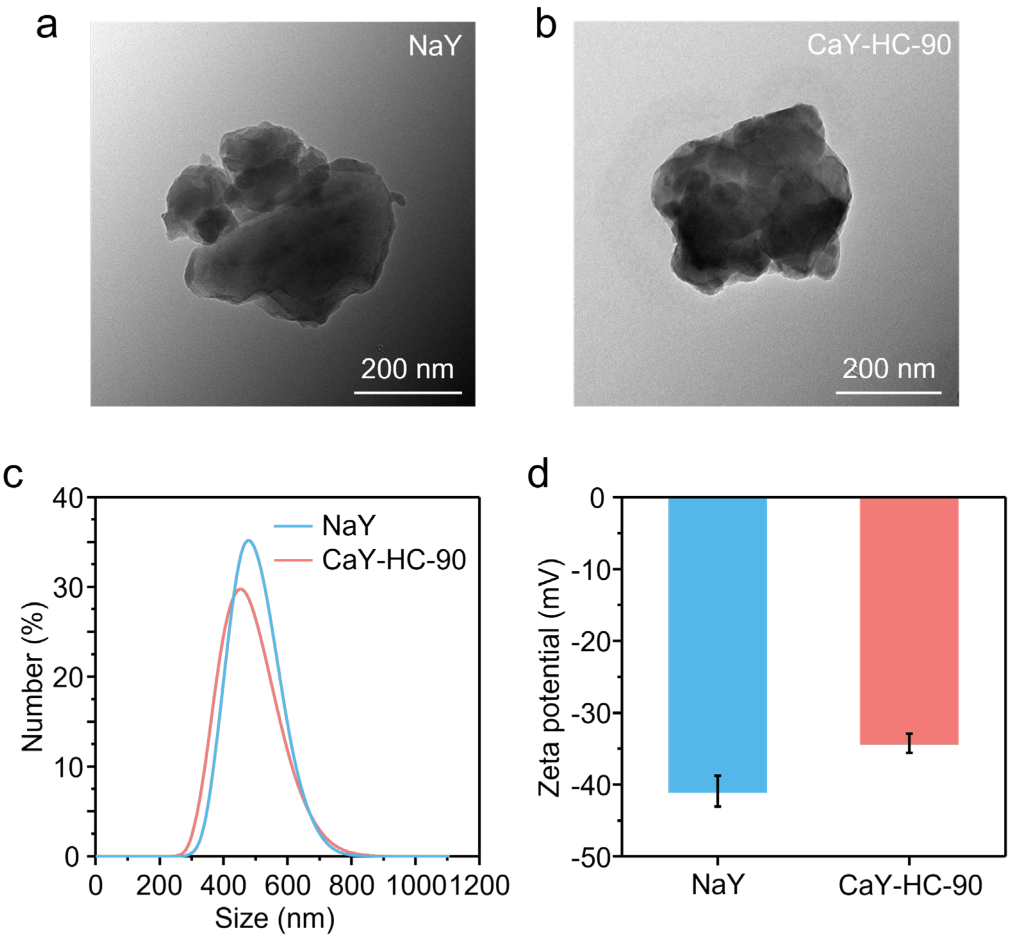

Figure S1. Characterizations of NaY and CaY-HC-90 zeolites. Transmission electron microscopy (TEM) images of (a) NaY and (b) CaY-HC-90 zeolites. Scale bar, 200 nm. (c) Size distribution of NaY and CaY-HC-90 zeolites. (d) Zeta potential of NaY and CaY-HC-90 zeolites (n=3). Data values correspond to mean ± S.D.

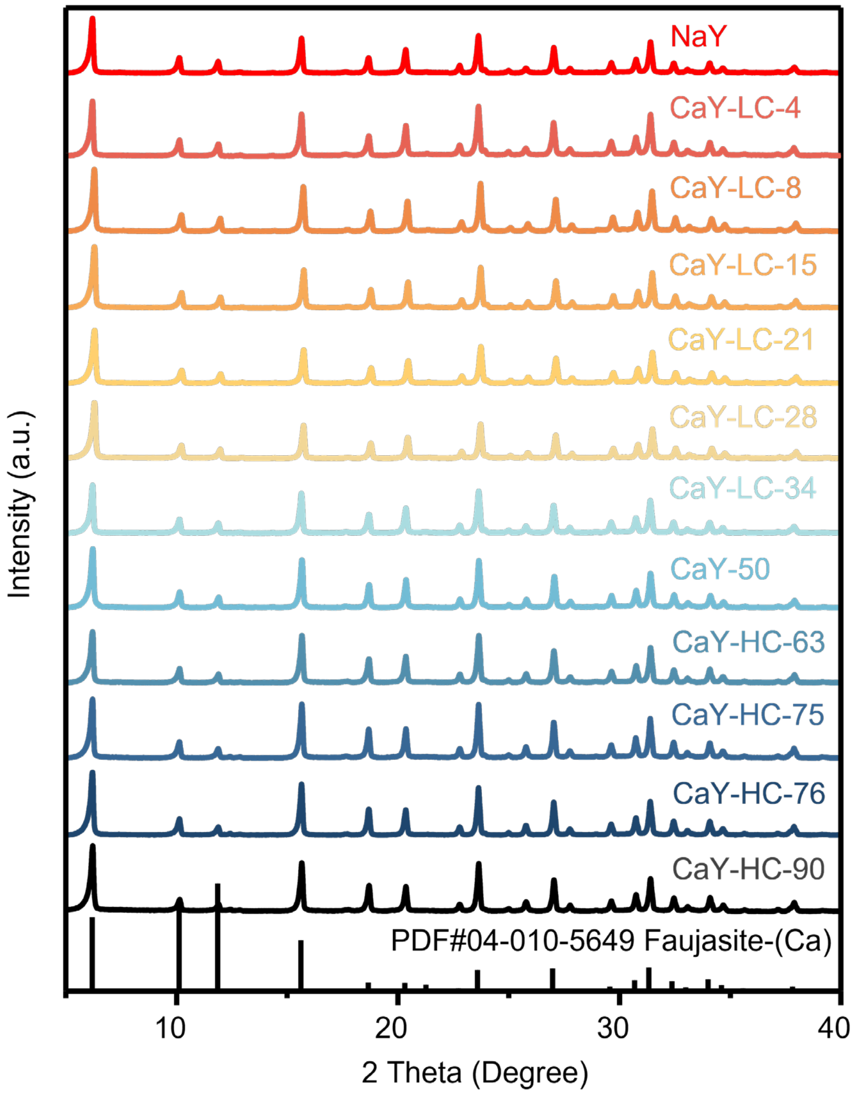

Figure S2. X-ray diffraction pattern (XRD) analysis of NaY and CaY zeolites.

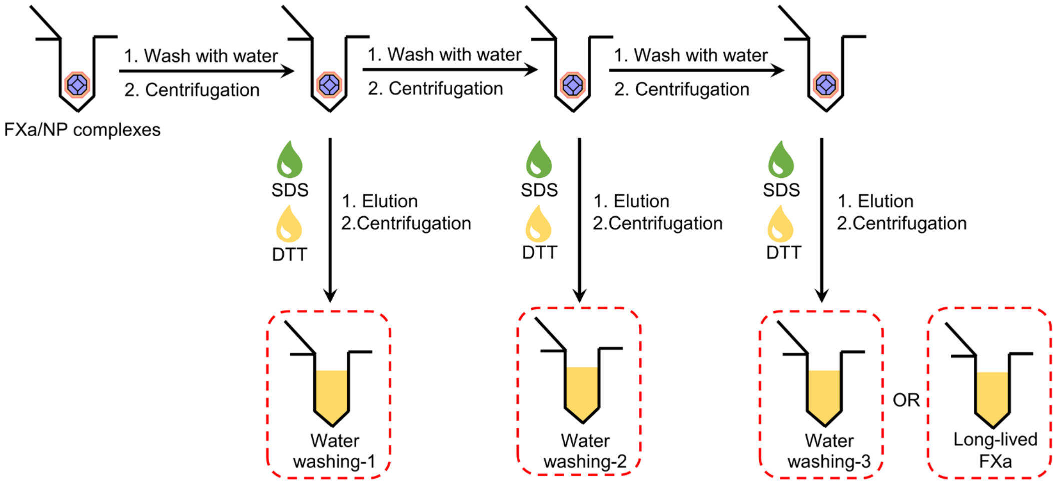

Figure S3. Work flow of separation experiment for shorted-lived FXa and long-lived FXa.

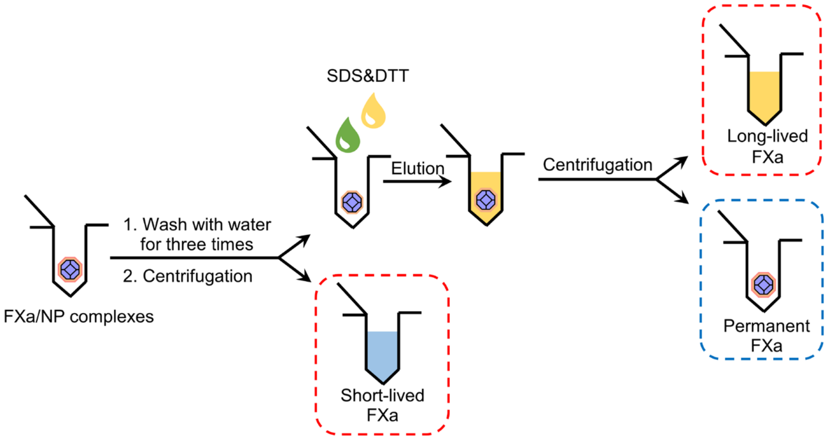

Figure S4. Work flow of separation experiment for shorted-lived FXa, long-lived and permanent FXa.

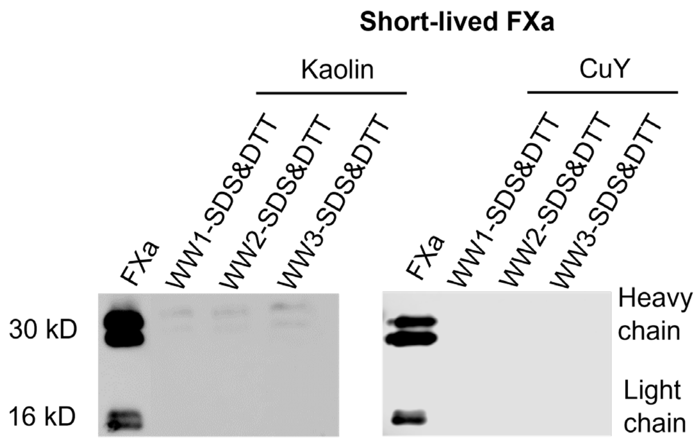

Figure S5. Short-lived FXa on kaolin and CuY zeolites identified by Western Blot (WB).

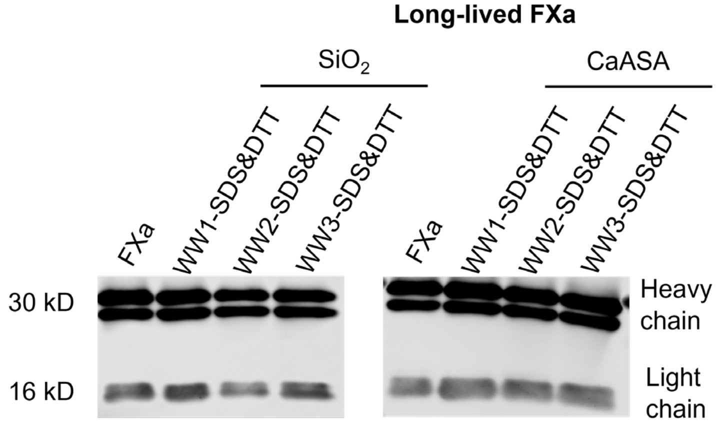

Figure S6. Long-lived FXa on SiO_2 and CaASA identified by WB.

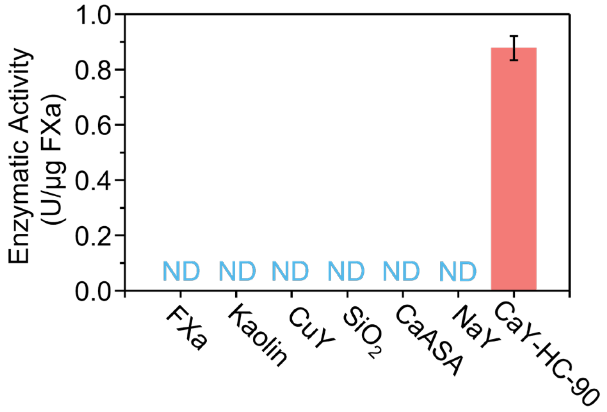

Figure S7. Specific enzymatic activity prothrombin-to-thrombin conversion of short-lived FXa on kaolin and CuY zeolites, long-lived FXa on SiO_2, CaASA, NaY zeolites, and permanent FXa on CaY-HC-90 zeolite (n=3). Data values correspond to mean ± S.D.

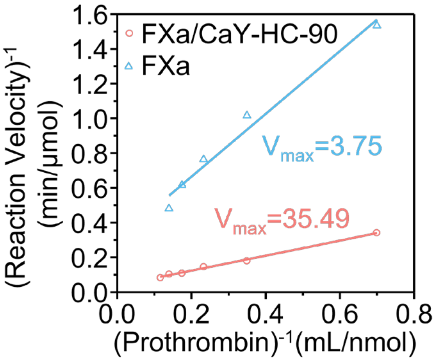

Figure S8. Michaelis-Menten constants of free FXa and FXa/CaY-HC-90 identified by Lineweaver-Burk plot. Unit of V_{max}: mol·min^{-1}·mol FXa^{-1}.

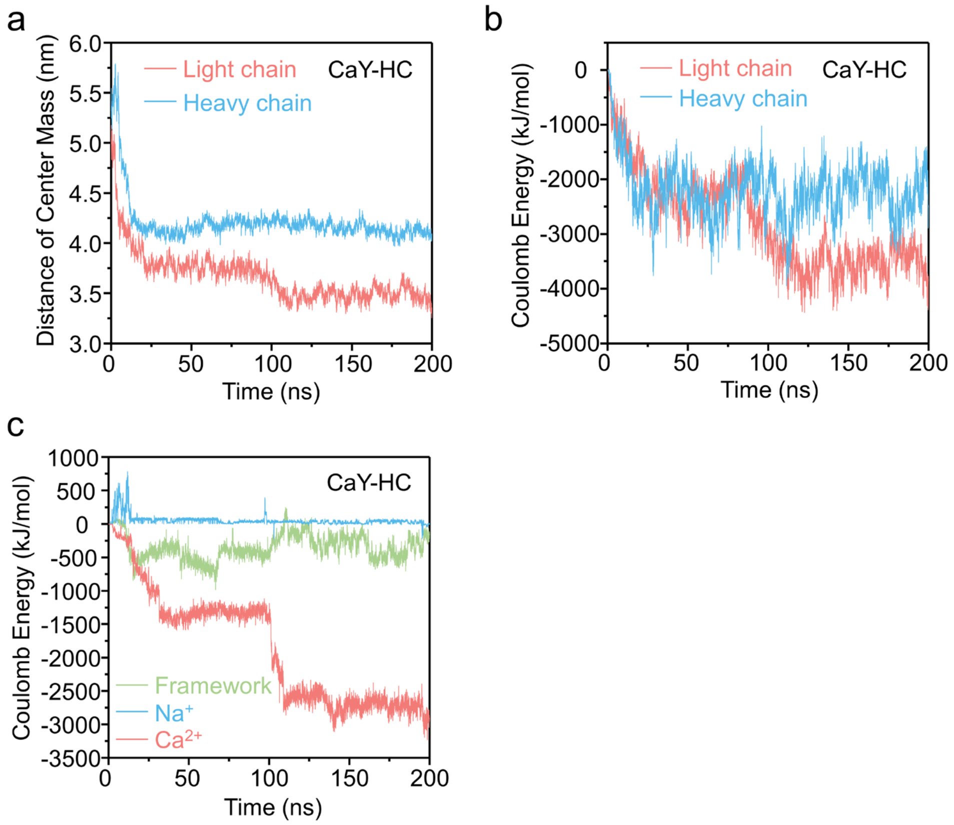

Figure S9. (a) Distance changing of center of mass (COM) of FXa heavy chain and FXa light chain while FXa adsorbing on CaY-HC zeolite. (b) Coulomb energy between FXa chains and Ca-HC zeolite. (c) Coulomb energy between FXa and components of CaY-HC zeolite.

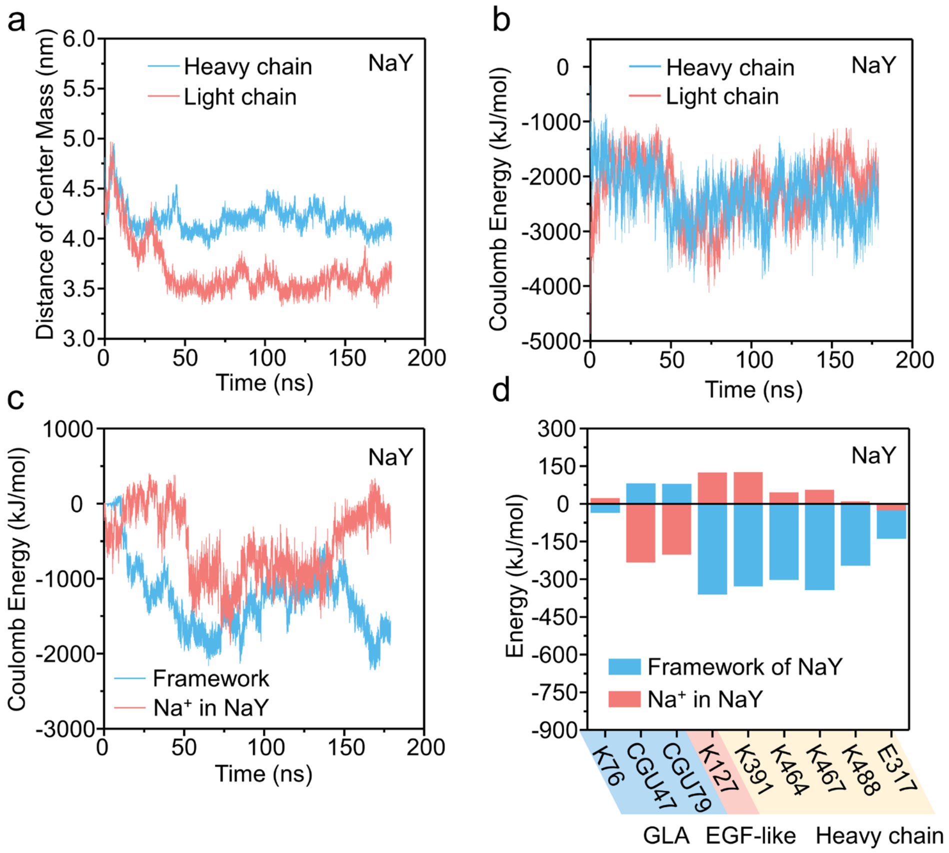

Figure S10. (a) Distance changing of center of mass (COM) of FXa heavy chain and light chain absorbed on NaY zeolite. (b) Coulomb energy between FXa chains and NaY zeolite. (c) Coulomb energy between FXa and components of NaY. (d) Binding energy between the main amino acid residues of FXa and NaY zeolite.

Table S6. Difference in glutamate benzoyl hydrazide labeling levels (FXa vs. FXa/CaY-HC-90)

		FXa			FXa/CaY-HC-90			
**Domain**	**CGU (E) sites**	**Peptide**	**Labeled peptide**	**Labeling level (%)**	**Peptide**	**Labeled peptide**	**Labeling level (%)**	**Delta value (%)**
GLA	CGU46	18	1	5.56	20	9	45.00	39.44
	CGU47	18	0	0	20	4	20.00	20.00
	CGU79	54	0	0	79	1	1.27	1.27
EGF-like 1	E91	70	55	78.57	7	1	14.29	-64.28
	E107	25	1	4.00	28	0	0	-4.00
	E114	101	6	5.94	106	1	0.94	-5.00
	E117	45	2	4.44	107	0	0	-4.44
	E122	536	34	6.34	595	2	0.34	-6.00
EGF-like 2	E142	38	2	5.26	28	0	0	-5.26
	E143	39	2	5.13	27	0	0	-5.13
	E178	24	13	54.17	43	0	0	-54.17
	Total	986	118	11.97%	1087	22	2.02%	-9.94

Table S7. Difference in lysine dimethyl labeling levels (FXa vs. FXa/CaY-HC-90)

Chain	K sites	FXa			FXa/CaY-HC-90			
		Peptide	Labeled peptide	Labeling level (%)	Peptide	Labeled peptide	Labeling level (%)	Delta value (%)
Light chain	K49	33	6	18.18	34	4	11.76	-6.42
	K50	26	4	15.38	17	2	11.76	-3.62
	K76	53	25	47.17	52	33	63.46	16.29
	K83	64	22	34.38	63	12	19.05	-15.33
	K85	53	28	52.83	98	45	45.92	-6.91
	K100	31	11	35.48	80	12	15.00	-20.48
	K102	66	49	74.24	27	15	55.56	-18.69
	K119	88	21	23.86	32	15	46.88	23.01
	K127	174	83	47.70	381	321	84.25	36.55
	K162	733	148	20.19	574	71	12.37	-7.82
	K174	811	237	29.22	614	289	47.07	17.85
Heavy chain	K242	87	82	94.25	24	12	50.00	-44.25
	K282	67	34	50.75	65	10	15.38	-35.36
	K310	995	926	93.07	961	530	55.15	-37.91
	K316	1314	1231	93.68	747	600	80.32	-13.36
	K329	388	231	59.54	273	151	55.31	-4.22
	K357	824	548	66.50	526	149	28.33	-38.18
	K370	197	140	71.07	179	65	36.31	-34.75
	K378	418	403	96.41	519	286	55.11	-41.31

K391	209	144	68.90	326	63	19.33	-49.57
K410	324	273	84.26	209	115	55.02	-29.24
K428	1273	476	37.39	1090	206	18.90	-18.49
K446	25	17	68.00	41	15	36.59	-31.41
K448	47	33	70.21	122	63	51.64	-18.57
K454	77	35	45.45	206	78	37.86	-7.59
K460	107	60	56.07	163	38	23.31	-32.76
K467	10	3	30.00	13	3	23.08	-6.92
K473	28	25	89.29	13	6	46.15	-43.13
K475	39	36	92.31	18	12	66.67	-25.64
Total	8598	5348	62.20	7496	3233	43.13	-19.07

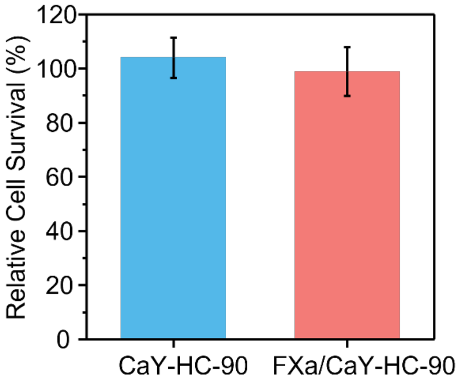

Figure S11. Cytotoxicity evaluation of L929 cells treated with CaY-HC-90 and FXa/CaY-HC-90-incubated media over 24 h. Data values correspond to mean ± SD, n= 4.

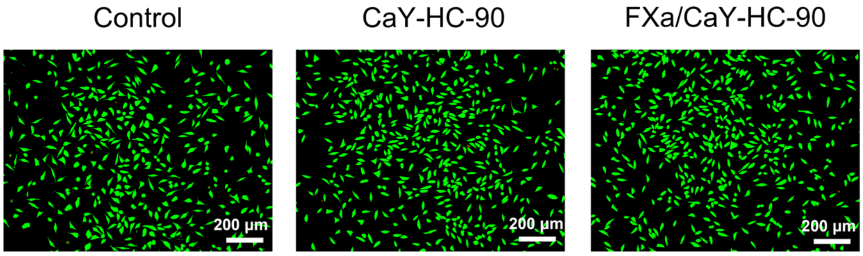

Figure S12. Live/dead staining results of L929 cells over 24 h in control, CaY-HC-90 and FXa/CaY-HC-90 groups, with living cells indicated in green and dead cells indicated in red.

Supplementary Reference

[1] E. A. Ruben, B. Summers, M. J. Rau, J. A. J. Fitzpatrick, E. Di Cera, *Blood* **2022**, *139*, 3463.

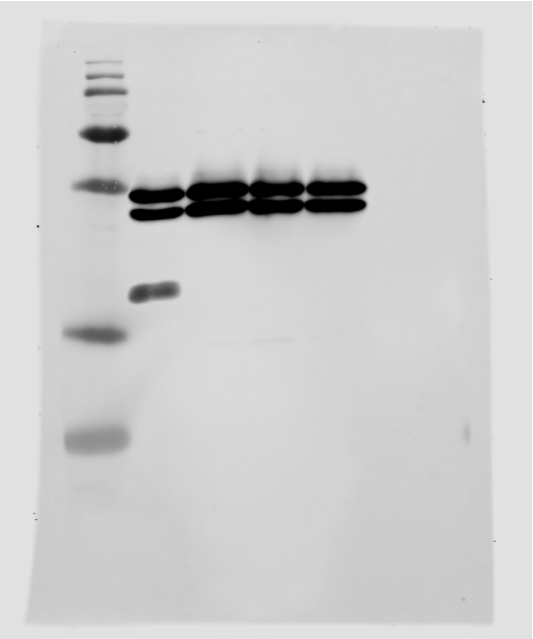

Additional Supplementary Figure 1. Uncropped Western Blots for Figure 1c.

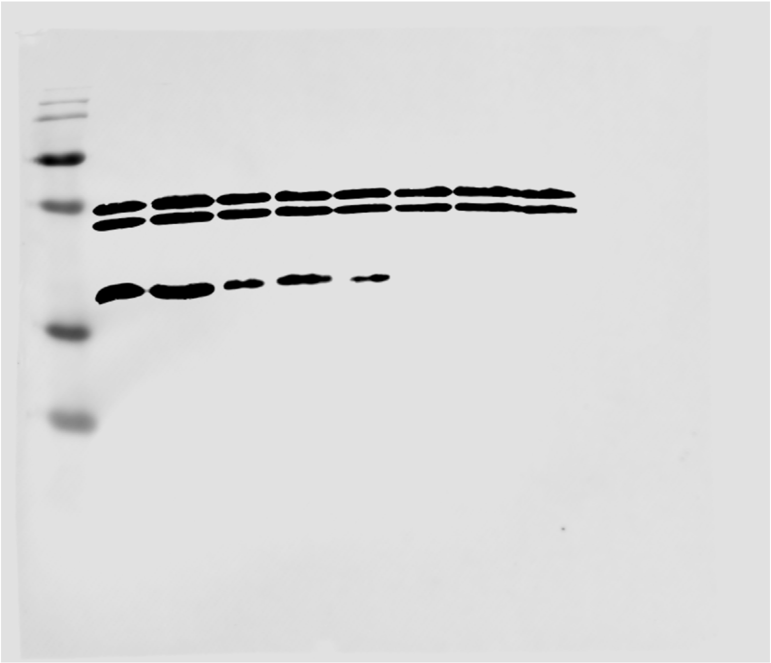

Additional Supplementary Figure 2. Uncropped Western Blots for Figure 1d.

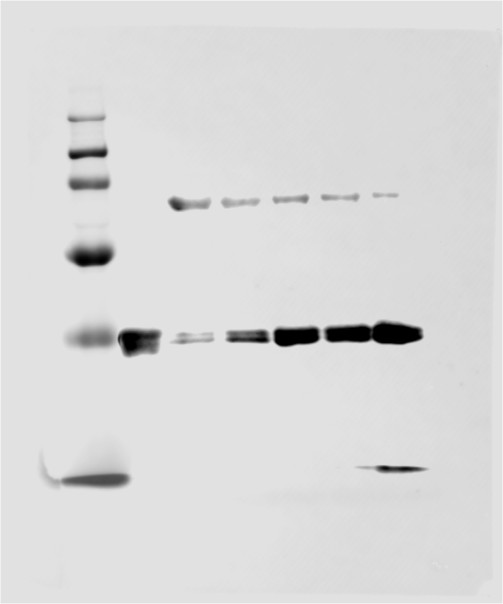

Additional Supplementary Figure 3. Uncropped Western Blots for Figure 1f.

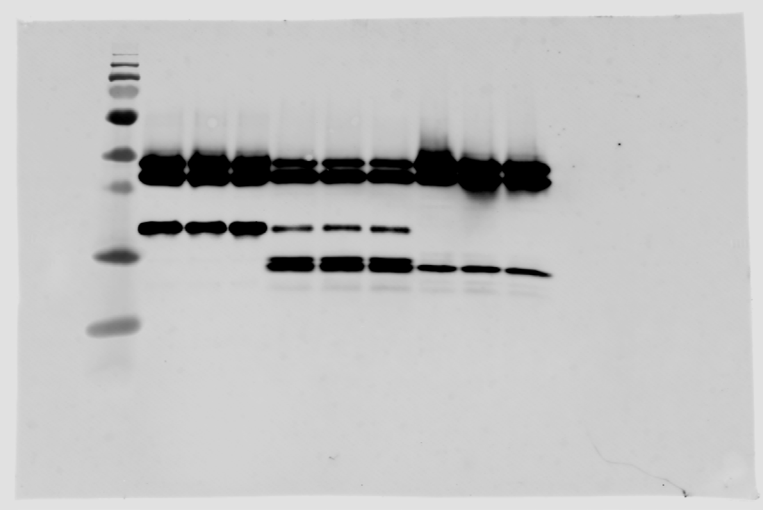

Additional Supplementary Figure 4. Uncropped Western Blots for Figure 1i.

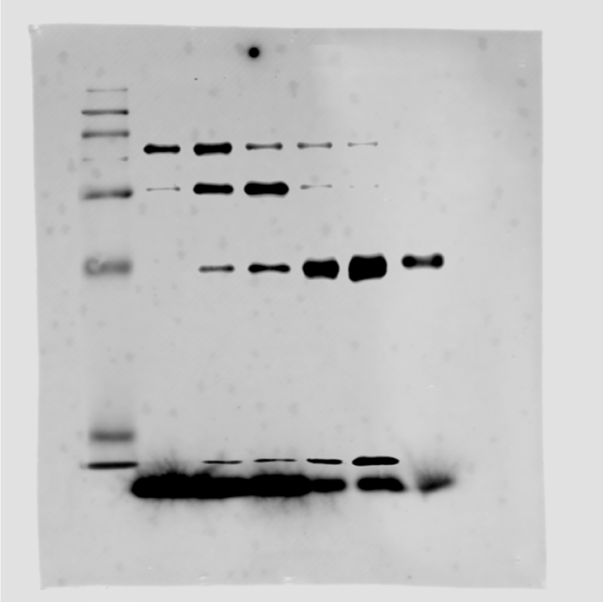

Additional Supplementary Figure 5. Uncropped Western Blots for Figure 4e.

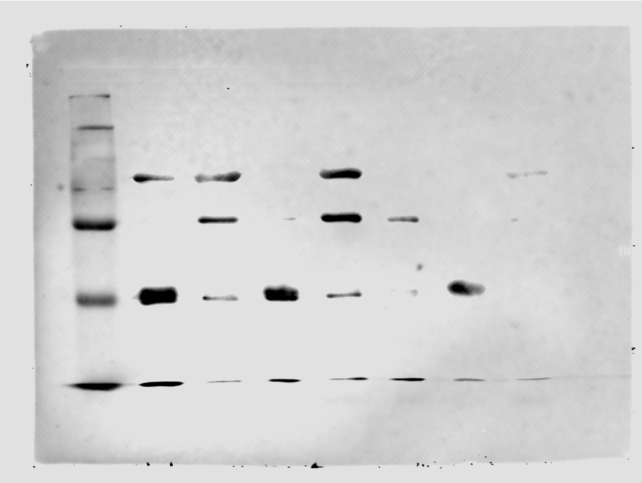

Additional Supplementary Figure 6. Uncropped Western Blots for Figure 4f.

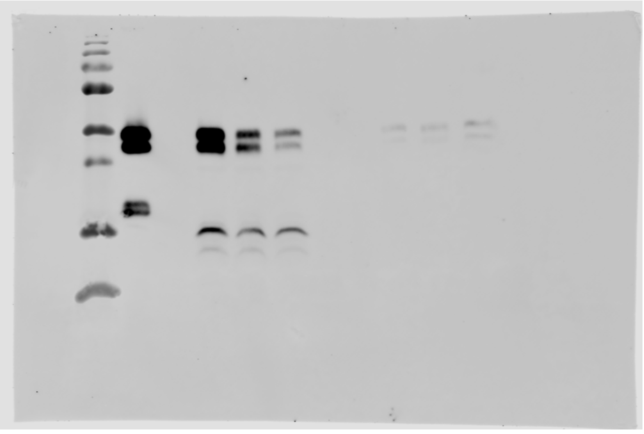

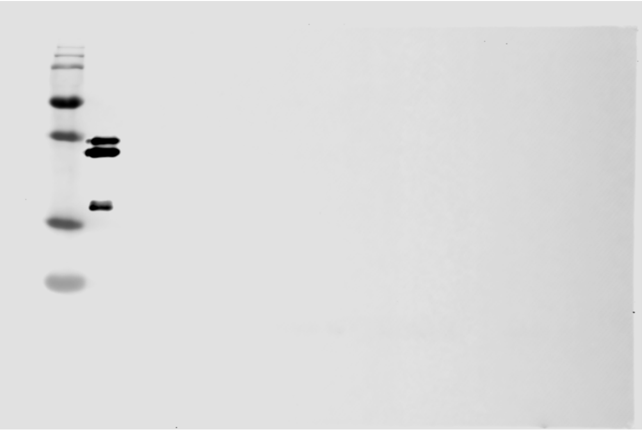

Additional Supplementary Figure 7. Uncropped Western Blots for Figure S5.

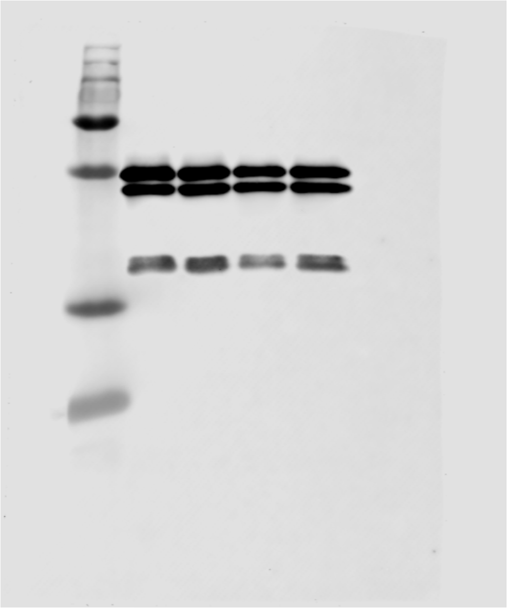

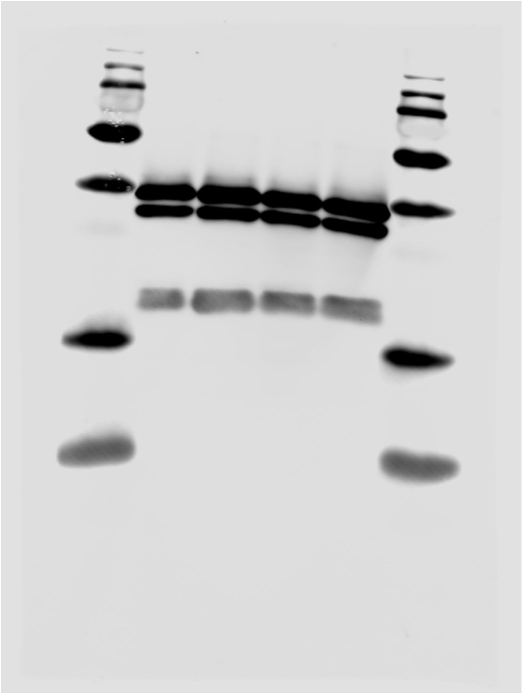

Additional Supplementary Figure 8. Uncropped Western Blots for Figure S6.
